# A randomized controlled trial on the use of pessary plus progesterone to prevent preterm birth in women with short cervical length (P5 trial)

**DOI:** 10.1186/s12884-019-2513-2

**Published:** 2019-11-27

**Authors:** Rodolfo C. Pacagnella, Ben W. Mol, Anderson Borovac-Pinheiro, Renato Passini, Marcelo L. Nomura, Kleber Cursino Andrade, Nathalia Ellovitch, Karayna Gil Fernandes, Thaísa Guedes Bortoletto, Cynara Maria Pereira, Maria Julia Miele, Marcelo Santucci França, Jose G. Cecatti, The P5 Working Group, Antônio F. Moron, Carlos A. S. Menezes, Claudio S. M. Paiva, Cristhiane M. Barros, Djacyr M. C. Paiva, Eduardo B. Alves, Elaine C. D. Moisés, Enoch Q. S. Barreto, Fernando M. Peixoto-Filho, Francisco E. L. Feitosa, Frederico Barroso, Herlanio C. Carvalho, Humberto S. Hirakawa, Julio Peña, Leila Katz, Marcelo C. C. Marques, Marcelo M. S. Lima, Marcos N. Pereira, Maria L. Costa, Maria T. P. Chaves, G. M. Marilia, Mário D. Correia-Jr, Nelson Sass, Renato L. S. Ximenes, Rodrigo P. S. Camargo, Rogério G. R. Guidoni, Rone P. C. Oliveira, Samira E. M. T. Haddad, Sérgio H. A. M. Costa, Silvana M. Quintana, Stéphanno G. P. Sarmento, Thais V. Silva

**Affiliations:** 1 Department of Obstetrics and Gynecology, School of Medical Sciences, University of Campinas – UNICAMP, Rua Alexander Fleming, 101 Cidade Universitária Zeferino Vaz, Campinas, 13087-460 Brasil; 2 Obstetrics & Gynaecology Monash Health, Monash University, Monash Medical Centre, 246 Clayton Road, Clayton, Victoria 3168 Australia; 3 Obstetric Unit, Woman´s Hospital, University of Campinas – UNICAMP, Rua Alexander Fleming, 101 Cidade Universitária Zeferino Vaz, Campinas, 13087-460 Brasil; 4 Ultrasound Department, Woman´s Hospital, University of Campinas – UNICAMP, Rua Alexander Fleming, 101 Cidade Universitaria Zeferino Vaz, Campinas, 13087-460 Brasil; 5 Federal University of São Paulo – UNIFESP, R. Napoleão de Barros, 715-Vila Clementino, São Paulo, SP 04024-002 Brasil

**Keywords:** Pessaries, Progesterone, Cervical length measurement, Randomized controlled trial

## Abstract

**Background:**

Preterm birth is the leading cause of mortality and disability in newborn and infants. Having a short cervix increases the risk of preterm birth, which can be accessed by a transvaginal ultrasound scan during the second trimester. In women with a short cervix, vaginal progesterone and pessary can both reduce this risk, which progesterone more established than cervical pessary. The aim of this study is to compare the use of vaginal progesterone alone versus the association of progesterone plus pessary to prevent preterm birth in women with a short cervix.

**Methods:**

This is a pragmatic open-label randomized controlled trial that will take place in 17 health facilities in Brazil. Pregnant women will be screened for a short cervix with a transvaginal ultrasound between 18 ^0/7^ until 22 ^6/7^ weeks of gestational age. Women with a cervical length below or equal to 30 mm will be randomized to the combination of progesterone (200 mg) and pessary or progesterone (200 mg) alone until 36 + 0 weeks.

The primary outcome will be a composite of neonatal adverse events, to be collected at 10 weeks after birth. The analysis will be by intention to treat. The sample size is 936 women, and a prespecified subgroup analysis is planned for cervical length (= < or > 25 mm).

Categorical variables will be expressed as a percentage and continuous variables as mean with standard deviation. Time to delivery will be assessed with Kaplan-Meier analysis and Cox proportional hazard analysis.

**Discussion:**

**I**n clinical practice, the combination of progesterone and pessary is common however, few studies have studied this association. The combination of treatment might act in both the biochemical and mechanical routes related to the onset of preterm birth.

**Trial registration:**

*Brazilian Clinical Trial Registry* (ReBec) RBR-3t8prz, UTN: U1111–1164-2636, 2014/11/18.

## Background

Preterm birth (PTB), defined as birth before 37 weeks of gestation, is in quantity and in severity one of the most important issue in obstetric care in the world. Globally, around 15 million preterm births are born every year [1]. In 2014 the United States it was 9.5% in 2014, while in Brazil, according to the national registry of living births system (SINASC), PTB rate was 9.9% in 2012 [2, 3]. The recent WHO report Born too Soon estimates the number of preterm births in Brazil to be 279,300 (9.1% of live births) [1]. Its prevalence has increased by 30% in the last 30 years and Brazil features among the top-10 countries with the highest numbers of preterm births [4]. Some data suggest that the real prevalence of preterm birth in Brazil approaches 10% [5].

Being born premature increases the risk of death by other causes as infections and is associated with lifelong sequelae such as cerebral palsy, visual deficiency and lower school performance [6]. Prematurity is the major cause of neonatal deaths worldwide and the second most important cause in children under 5 years, after pneumonia. Only in 2010 prematurity was responsible for 35% of 3.1 million neonatal deaths [4].

PTB is a multifactorial condition. The increase in maternal age at conception, multiple pregnancies, and maternal and neonatal related conditions are some of these factors that lead to therapeutic and spontaneous preterm labor. Women with previous PTB, preterm premature rupture of membranes (PPROM), and preterm labor also have a higher risk of PTB [7]. Even knowing some of the causes, almost 50% of the spontaneous PTB have no identifiable cause [7] and a significant reduction of PTB rates has not been accomplished so far.

One raising strategy to reduce PTB is to pay attention to physiological modifications that precede preterm labor in the uterine cervix: the cervical length shortening [8]. There is an inverse relationship between mid-trimester cervical length and the risk of preterm birth that varies as a function of the gestational age at the time the cervical shortening is diagnosed [6].

The process of cervical effacement begins some weeks before preterm labor and cervical shortening diagnosed between the 16th and 24th weeks is an important etiological factor for preterm delivery [9]. This has been demonstrated in populations with different risk profiles, varying from asymptomatic women with low-risk singleton pregnancies to women with high-risk pregnancies due to previous preterm birth or a twin pregnancy [10–12].

The best way to identify cervical shortening is by using transvaginal ultrasound examination performed between the 16th and 24th weeks of gestation. Although the cut-off limit for the cervical length that represents a risk for preterm delivery is subject to debate, the relative risk of preterm delivery among women with cervical length below the 25th percentile (30 mm) was shown to be at almost 4 times higher than for those above 40 mm [13].

Today, strategies to predict PTB include sonographic measurement of the cervical length. The shortening of cervical length is a common pathway leading to preterm labor. The shorter the cervix, the greater the risk of PTB, therefore, there may be an important role to screen for risk of preterm birth using cervical shortening [8]. Cervical length measure is best accessed by transvaginal ultrasound scan between 16 and 24 weeks and can be performed along with the routine second-trimester ultrasound [14].

When a short cervix is diagnosed during the second trimester, maternal use of progesterone reduces the rate of PTB with 30–45% [15–17]. A meta-analysis [15] indicated progestational agents generated lower rates of preterm delivery in women with a short cervix (26.2% vs 35.9%; OR 0.45, 95% CI 0.25–0.80). Relative to women allocated to receive placebo, those who received progestational agents also had lower rates of perinatal mortality (14.8% versus 17.1%; OR 0.69, 95% CI 0.38–1.3).

Progesterone can be used as either intramuscular (17 alpha-hydroxyprogesterone caproate 250 mg), subcutaneous (progesterone 25 mg), vaginal gel (progesterone 80 mg) or vaginal capsules (micronized progesterone 200 mg) but there is some evidence that vaginal progesterone is superior to other presentations to prevent PTB [18], and seems to be safe for the mother and the fetus [19]. Twin pregnancies have also a higher risk of PTB, but progesterone is not effective in unselected women with a twin pregnancy. However, the use of progesterone in patients with twin gestation and short cervix seems promising [20].

Another promising intervention to prevent preterm delivery is the use of a cervical pessary. The pessary seems to exert its effect through mechanically changing the cervico-uterine angle, reducing the compression of the cervix and protecting the cervical mucus [21]. Studies in both twins and singletons have indicated that the cervical pessary may be an effective intervention in the prevention of spontaneous preterm birth and the harm resulting from it.

Although some studies have failed to demonstrate benefits of the pessary for PTB both in singletons and twins [22–26], others [27–30] have shown a significant reduction in PTB rate in women with short cervix using a pessary. The PECEP trial was the first well-conducted randomized control trial [27] showing that in women with a short cervical length, the use of a cervical pessary reduces the preterm birth rate.

In summary, treatment with both pessary and progesterone might be helpful in reducing the rate of preterm birth. Both interventions have shown to be safe and reliable, as no maternal or fetal severe outcomes due to the use of these interventions have been demonstrated. However, the combination of progesterone and pessary for the prevention of PTB has not been tested yet. A combination could guide both pathways: the biochemical (progesterone) and mechanical (pessary), making the combined treatment more effective than a single treatment, acting in a broader set of women at risk for PTB [31, 32].

The aim of this study is to compare the effectiveness of the use of progesterone alone versus the combination of progesterone plus pessary to prevent PTB in women with short cervical length.

## Methods

This is a pragmatic, multicentre, open-label randomized controlled trial (RCT), testing the effectiveness of pessary plus progesterone versus progesterone alone in reducing preterm birth and a composite of neonatal adverse events.

### Governance

The coordinating center of the study is the Women’s Hospital at the University of Campinas in Brazil. There are 17 reference obstetric units in different geographic regions of Brazil as participating centers, including academic and general maternity hospitals and other health facilities such as ultrasound clinics and prenatal care units. The study participating centers are all members of the *Brazilian Network for Studies in Reproductive and Perinatal Health*. Each center has a local investigator, who may also select research assistant medical doctors to proceed with the intervention.

The study will be overseen by a Steering Committee (SC-P5) and a Data Safety and Monitoring Board (DSMB-P5). The SC-P5 is composed of the principal investigators, researchers of recognized national and international competence, a representative of an NGO dealing with preterm birth patients and members of the funding agencies (Bill & Melinda Gates Foundation, National Council for Scientific and Technological Development (CNPq) Ministry of Health) to evaluate the progress of the study and suggest changes when necessary.

The DSMB-P5 is composed of researchers of recognized national and international competence without any participation in the study group with the support of a statistician. This committee has complete access to the database and will meet at prespecified moments.

The Brazilian National Review Board (CONEP) approved the study under the number 1.055.555. Each local Institutional Review Boards also approved the study protocol. Protocol modifications will be reported to each relevant part including national and local institutional review boards and will be available to trial participants through the project website, to the investigators by an internal bulletin, to the registries and journals by publications, and to regulators by the review board report. No funding agency had any influence on the design and decision regarding data collections, managements, analysis and writing.

### Trial organization

A Standard Operation Procedure with video classes was developed, provided to all centers and available online, with information on how to collect and store data, how to proceed with randomization, follow-up, and outcomes. The number of subjects will be monitored weekly by the project coordinators, monitoring visits to the other participating centers twice a year. The reports from this monitoring process will be available to the monitoring committees and to the centers themselves to document the need for improvement.

The research assistant will collect informed consent before submitting the participant to the ultrasound scan, and in case of the short cervix, another consent will be applied before the randomization. Data will be kept confidential during the study. The name and the personal information will be stored in a database different from the main results and will be available only to each respective local coordinator during the follow-up. After the end of the study personal data will be split from the clinical results and will be available only to the principal investigator (PI).

**Fig. 1:**
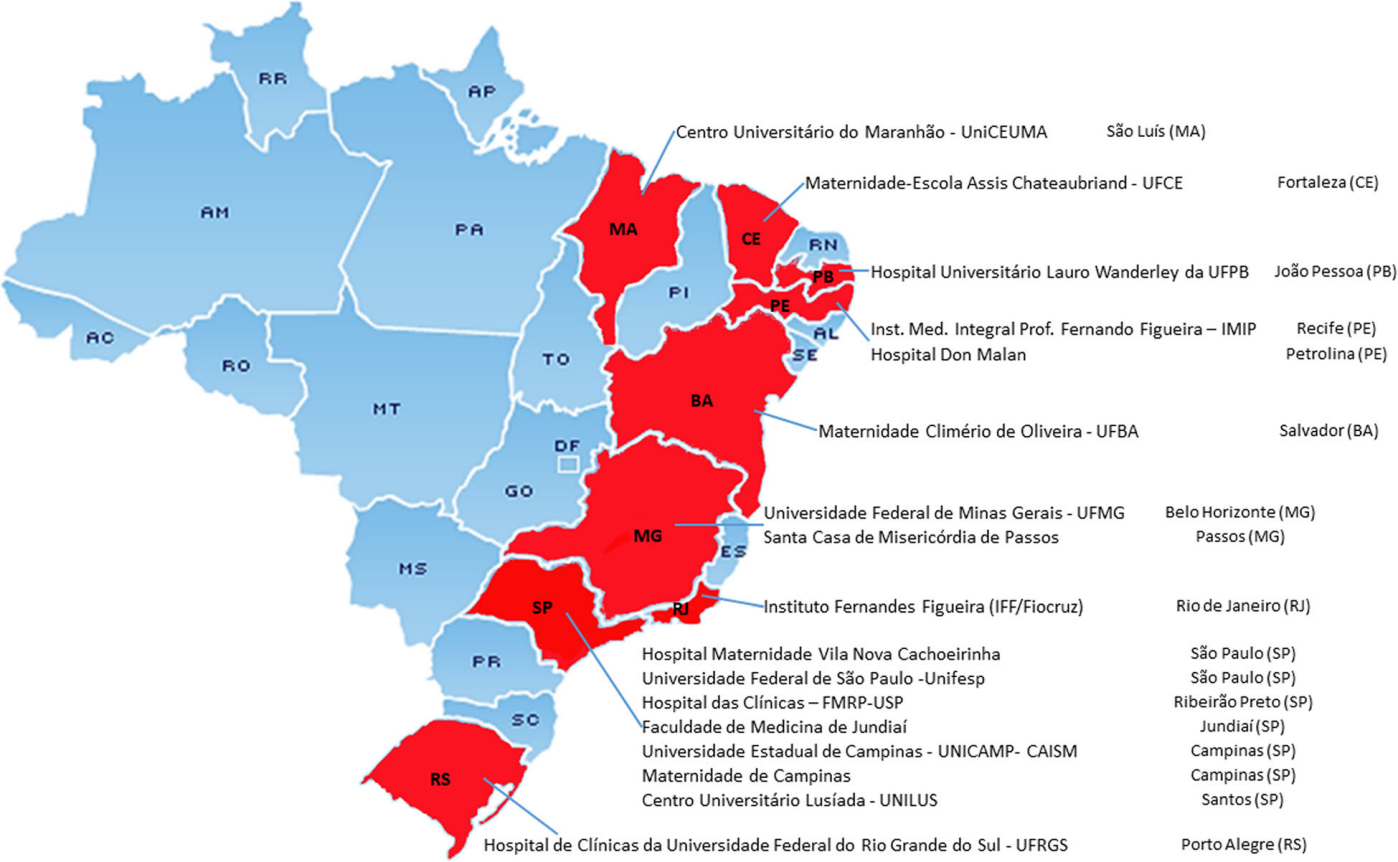
Participating centers and location

The final dataset will be available to the PI and collaborating researchers. If necessary, participants will receive ancillary and post-trial care, as compensation if there is any harm secondary to the participation on the trial. The PIs do not have any competing interests for the trial. Trial results will be published as scientific articles, and authorship eligibility will be considered according to the *International Committee of Medical Journal Editors* recommendations. Participating centers are listed in Fig. 1.

### Inclusion and exclusion criteria

We will study women with a singleton or twin pregnancy. Pregnant women, no maternal age restriction, at a gestational age between 18 ^0/7^ and 22 ^6/7^ weeks will be offered cervical length measurement by ultrasound (US). Women with a cervical length below or equal to 30 mm (but more than 5 mm) are eligible for the trial.

Exclusion criteria are painful contractions, vaginal bleeding, cerclage during current pregnancy before the screening, PPROM, severe liver disease, cholestasis during this pregnancy, previous or current thromboembolism, placenta previa, cervical dilation greater than 1 cm, monoamniotic twin pregnancy, higher order twin pregnancy (triplets or higher), major fetal malformation of at least one fetus and stillbirth.

### Ultrasound training

All participating members of the research team are trained to perform the cervical measurement by ultrasound and to manage the pessary. All sonographers are trained in cervical measurement according to The Fetal Medicine Foundation training program [33] and are encouraged to follow its certification program. Additionally, an online training program (using Moodle platform) was created to certificate all sonographers involved in the P5 study in standardizing cervical measurements, and at this Moodle platform, all participants of the research had signed the disclosure of contractual agreements that limit such access for investigations. The ultrasound examination will be performed using a GE Logic C5® equipment.

### Recruitment and enrolments

A research assistant will invite women attending the ultrasound department of each institution or outpatient clinics involved in the study to participate in the screening strategy and, after being informed about the study, to sign a written Informed Consent Form. A transvaginal ultrasound scan to measure cervical length as described above will be performed.

Women with a short cervix and without any exclusion criteria will then be invited to participate in the randomized clinical trial. After being informed about the study verbally and with a written patient information leaflet, women will be asked to sign an Informed Consent Form. Only after written informed consent, the local investigator will randomly assign patients. In case of an eligible participant under 18 years old, a consent form will be signed for both the underaged participant and his/her legal guardian/parent before been admitted into the study.

### Randomization

After written informed consent, women will be randomized into two groups: the use of pessary plus progesterone or the use of progesterone alone. Randomization will be stratified by center, number of fetuses (one or two) and cervical length (< 25 mm or 25-30 mm) using a 1:1 ratio and variable block sizes. Randomization is performed electronically in an online database added as a specific form in the online data collection system, using computer-generated random numbers. The system can be accessed 24/7 and a phone number is available to help the inclusion and randomization process. Each local coordinator will be responsible for the randomization process including enrollment and assignment of women to interventions.

### Interventions

Since the use of the pessary cannot be blinded, this is an open-label RCT. The pessary used is an Arabin type pessary registered at the national health surveillance agency of Brazil (Pessário AM® by Ingamed) with the dimensions: inner circle 30 mm; outer circle 70 mm; height 25 mm.

A physician trained specifically for pessary placement will insert the pessary up to 72 h from the randomization. An ultrasound scan to re-assess cervical length and pessary placement will be performed after its insertion.

We will use 200 mg micronized progesterone capsules that will be self-administered digitally inserted into the vagina every night. Commercial Progesterone (Utrogestam®) pills will be purchased from Besins Healthcare Pharmaceutics. Both interventions will be maintained until 36 weeks gestation unless antenatal care providers consider the need to withdrawal earlier.

The intervention will be discontinued before 36 weeks only if the assistant medical doctor judges it is necessary due to clinical conditions, or in case of woman request. Every enrolled woman will receive access to a P5 phone number for her to call in case of doubts or need to report side events. A research assistant will be full time available for solving such questions.

Women with threatened preterm labor will be treated with tocolysis, corticosteroids and magnesium sulfate according to local protocols.

### Follow-up

Prenatal care will continue in each reference facility considering local protocols. In each visit, the participant will be asked about the use of progesterone and pessary, symptoms, adverse events, and admission to hospital. She will receive enough progesterone until the next appointment when more capsules will be delivered. One research assistant per center will follow up all enrolled women avoiding double data collection. Adherence monitoring will be checked in all visits with the woman returning her drug table.

All enrolled women will be followed-up until 10 weeks after birth. To avoid a loss to follow-up, participants will be contacted by telephone if they miss an appointment. Compliance will be measured by the number of progesterone pills returned by the subject at each antenatal care visits.

### Outcomes

Our primary outcome is a composite of neonatal adverse events: periventricular leukomalacia (focal periventricular necrotic and diffuse gliosis in the surrounding cerebral white matter that may have cystic lesions secondary to necrotic foci in the white matter) [34], severe respiratory distress syndrome (grunting, retractions, or other typical distress symptoms in a premature infant immediately after birth with a chest radiography showing homogenous opaque infiltrates and air bronchograms) [35], bronchopulmonary dysplasia (infant requiring treatment with > 21% oxygen for at least 28 days) [36], periventricular hemorrhage grade II (hemorrhage is present in a nondistended lateral ventricle) or higher [37], necrotizing enterocolitis (spectrum of intestinal conditions including feeding intolerance, mild abdominal distention, spontaneous intestinal perforations and intestinal necrosis) [38], proven sepsis before discharge (generalized bacterial infection occurring within the neonatal period up to 4 weeks beyond the expected date of delivery accompanied by a positive blood culture) [39], stillbirth, or neonatal death.

Secondary outcomes will be gestational age at birth, birth weight, adequacy of birth weight for gestational age, first and fifth minute Apgar score, neonatal gestational age, overall, spontaneous and medical induced PTB rate before 28, 32, 34 and 37 weeks of gestation, PPROM rate, neonatal intensive care unit (NICU) admission and length of stay, maternal morbidity and mortality, maternal length of stay in hospital, maternal ICU admission, cost-benefit analysis of the screening program, treatment cost, maternal inpatient treatment costs, neonatal inpatient treatment costs and pessary use unwanted effects [40, 41].

Information on readmissions of mother and/or newborn and primary and secondary outcome will be collected during hospitalization for birth and at 10 weeks after due date, by telephone contact and patient’s registries review.

### Sample size

Considering the reduction in the risk of preterm delivery by the pessary described by the PECEP clinical trial [27], and considering our own outcomes in women with a short cervix, we anticipate a composite poor neonatal outcome rate of 22%. We consider a reduction of 10% as clinically relevant. The reduction from 22 to 12% in the composite poor neonatal outcomes (2-sided alpha 0.05, 80% power) can be detected if 438 women are randomized (219 per group). To assure a subgroup analysis for cervical length less than or equal to 25 mm, considering a reduction of 50% in adverse neonatal outcomes (17.3 to 8.65% [15]), it will be necessary to increase a sample to 468 women (234 per subgroup). Thus, the final sample should be composed of 936 women (468 in each group, 234 per subgroup).

### Data collection

The data will be collected using an online database, which is a new generation of application systems that allow the collection and cleaning of clinical trial data using the Internet. This technology was developed specifically for the study using an existing certified platform for medical offices management. The online database has distinct permission levels and is encrypted and protected by a personal security password. Data will be stored in cloud network and a backup is being performed every day.

The forms and the randomization process are inserted in the platform and access is given to each facility coordinator and research assistant for local data management. Each local coordinator is responsible for data entry. Data will be collected in printed forms or directly in the online form, available in the same online database where the randomization takes place, by a trained research assistant. The local coordinator will verify these data in the printed and online system, to assure the information is correct.

### Data analysis

Data analysis will be performed according to the intention-to-treat principle. A statistical analysis plan will be finalized prior to the data lock.

Categorical variables will be expressed by the relative frequency on the corresponding allocation arm. For continuous variables minimum, first quartile, median, third quartile, maximum, mean, and standard deviation will be presented. Missing values will be reported for all variables.

The primary outcome is a binomial random variable. For this outcome, we will estimate a risk ratio (RR) with 95% CI. We will also calculate rates in the two groups as well as the number needed to treat (NNT). The comparisons of treatments will be performed with a generalized linear model [42], with the log link function. The model will include treatment as main effect and a full factorial of study center, type of pregnancy (twin or singleton) and cervix length (two categories). The main analysis will be performed using stratums according the randomization process. All factors are fixed. The factors study center, type of pregnancy and cervix length figure as randomization block terms.

An additional model including cervical length as continuous covariate will also be analyzed. The model better adjusted will be used as final model. The Akaike Information Criterion (AIc) e Bayesian Information Criterion (BIC) will be used to decide among models. To evaluate the potential efficacy of the pessary, a per-protocol analysis will also be performed.

Secondary outcomes will be analyzed using generalized linear models with treatment as main effect and a full factorial of study center, type of pregnancy (twin or singleton) and cervix length (two categories). All factors are fixed. An appropriated link function will be chosen to each response variable.

An additional model including cervical length as continuous covariate will also be analyzed. The model better adjusted will be used as final model. The Akaike Information Criterion (AIc) e Bayesian Information Criterion (BIC) will be used to decide among models.

Survival analysis to assess time to delivery will also be performed using Kaplan Meier and Cox Proportional Hazard Model. Differences in treatments among subgroups will be assessed including in the statistical models already described above a subgroup variable and a interaction term subgroup-treatment. These are the subgroups to be analyzed:

- Singleton and twin pregnancies
- Cervical length (≤25 mm / > 25 mm)
- Nulliparous/ multiparous
- Previous spontaneous preterm birth
- Obstetric US abnormalities or sludge at randomization
- Ethnicity (white / non-white)

The DSMB-P5 will meet at predetermined intervals (after half of the randomized cases and after 2/3 of the randomized cases) to analyze the results regarding the possible adverse effects of the interventions or regarding the achievement of statistical significance for the main outcome. Other interim analyses will be calculated according to DSMB-P5 request. The committee will have the power to recommend completion of the study or an early interruption based on the evaluation of the interim results and on some pre-specified stopping rules.

### Timetable

The study started woman enrolment in July 2015. Chronogram considers finishing enrolment in April 2019. Final reports and publication are expected by December 2019.

### Registration and version

The study protocol was registered in the *Brazilian Clinical Trial Registry* (ReBec) on November 18th, 2014 and the register number is RBR-3t8prz UTN: U1111–1164-2636. This is the version 7 of the protocol. This manuscript adhered to SPIRIT guidelines/methodology.

## Discussion

Preterm birth is still an important condition in obstetrics; however, a significant reduction of PTB rates has not been accomplished so far. It is a complex syndrome involving inflammatory agents and epigenetic factors and few strategies have being proven to reduce the risk of preterm birth, being the use of progesterone the most effective intervention for preventing PTB in singleton pregnancy [43, 44].. Cervical pessary have also shown a role in the reduction of spontaneous preterm birth at < 37 weeks, without negatively affecting maternal or neonatal outcomes [45].

Although in clinical practice the combination of progesterone and pessary is common, few studies have studied the association of treatments in the reduction of preterm birth [46]. A previous cohort study showed that the association of cervical pessary and vaginal progesterone did not reduce the risk of PTB as compared to the use of the pessary alone [32]. Another study suggested that the combination cervical pessary plus daily vaginal progesterone does not reduce the rate of preterm birth compared with vaginal progesterone alone [47]. However these studies are small and susceptible to bias, therefore, more studies are needed [46].

Treatment with both pessary and progesterone might work in synergy acting in both the biochemical (progesterone) and mechanical (pessary) routes related to the onset of preterm birth [31, 32]. We believe that the present trial may offer high-level evidence for the prevention of PTB.

## Supplementary information

**Additional file 1.** Participating centers and location in Brazil.

## Data Availability

Not applicable.

## Abbreviations

(CNPq) Ministry of Health: National Council for Scientific and Technological Development
CONEP: Brazilian National Review Board
DSMB-P5: Data Safety and Monitoring Board
NICU: Neonatal intensive care unit
NNT: Number needed to treat
PI: Principal investigator
PPROM: Preterm premature rupture of membranes
PTB: Preterm birth
RCT: Randomized controlled trial
ReBec: *Brazilian Clinical Trial Registry*
SC-P5: Steering Committee
SINASC: National registry of living births system
US: Ultrasound
