# Supplementary material for: A randomized controlled trial on the use of pessary plus progesterone to prevent preterm birth in women with short cervical length (P5 trial)
Source: BMC Pregnancy Childbirth. 2019 Nov 27;19:442. doi: 10.1186/s12884-019-2513-2 (PMC6880495; doi:10.1186/s12884-019-2513-2)
Supplement: Supplementary file 1 — Additional file 1. Participating centers and location in Brazil. [file 12884_2019_2513_MOESM1_ESM.doc]

Participating centers and location in Brazil

| **Facility** | **Location** | **Ethics Committee name** |
| --- | --- | --- |
| 1. Centro Universitário do Maranhão - UniCEUMA | São Luís (MA) | 5084 - Centro Universitário do Maranhão - UniCEUMA |
| 1. Maternidade-Escola Assis Chateaubriand - UFCE | Fortaleza (CE) | 5050 - UFC - Maternidade Escola Assis Chateaubriand da Universidade Federal do Ceará / MEAC - UFC |
| 1. Hospital Universitário Lauro Wanderley da UFPB | João Pessoa (PB) | 5183 - UFPB - Hospital Universitário Lauro Wanderley da Universidade Federal da Paraíba |
| 1. Instituto de Medicina Integral Professor Fernando Figueira – IMIP | Recife (PE) | 5201 - Instituto de Medicina Integral Professor Fernando Figueira - IMIP/PE |
| 1. Hospital Don Malan | Petrolina (PE) | 5201 - Instituto de Medicina Integral Professor Fernando Figueira - IMIP/PE |
| 1. Maternidade Climério de Oliveira - UFBA | Salvador (BA) | 5543 - UFBA - Maternidade Climério de Oliveira |
| 1. Universidade Federal de Minas Gerais - UFMG | Belo Horizonte (MG) | 5149 - Universidade Federal de Minas Gerais |
| 1. Santa Casa de Misericórdia de Passos | Passos (MG) | 8043 - Santa Casa de Misericórdia de Passos |
| 1. Instituto Nacional de Saúde da Mulher, da Criança e do Adolescente Fernandes Figueira (IFF/Fiocruz) | Rio de Janeiro (RJ) | 5269 - Instituto Fernandes Figueira - IFF/ FIOCRUZ - RJ/ MS |
| 1. Maternidade de Campinas | Campinas (SP) | 5404 - UNICAMP - Campus Campinas |
| 1. Hospital Maternidade Vila Nova Cachoeirinha | São Paulo (SP) | 5454 - Hospital Municipal e Maternidade-Escola Dr. Mário de Moraes Altenfelder Silva |
| 1. Hospital das Clínicas da Faculdade de Medicina de Ribeirão Preto -USP | Ribeirão Preto (SP) | 5440 - USP - Hospital das Clínicas da Faculdade de Medicina de Ribeirão Preto da USP - HCFMRP/USP |
| 1. Faculdade de Medicina de Jundiaí | Jundiaí (SP) | 5412 - Faculdade de Medicina de Jundiaí |
| 1. Universidade Federal de São Paulo -Unifesp | São Paulo (SP) | 5505 - UNIFESP - Hospital São Paulo - Hospital Universitário da Universidade Federal de São Paulo - HSP/UNIFESP |
| 1. Universidade Estadual de Campinas - UNICAMP- CAISM | Campinas (SP) | 5404 - UNICAMP - Campus Campinas |
| 1. Centro Universitário Lusíada - UNILUS | Santos (SP) | 5436 - Centro Universitário Lusíada / Fundação Lusíadas |
| 1. Hospital das Clínicas da Universidade Federal do Rio Grande do Sul - UFRGS | Porto Alegre (RS) | 5327 - UFRGS - Hospital de Clínicas de Porto Alegre da Universidade Federal do Rio Grande do Sul / HCPA |
